# Treatment Patterns and Outcomes of Patients With Myelodysplastic Syndrome (MDS) by IPSS‐R Scores at Academic Cancer Centers

**DOI:** 10.1155/ah/9924808

**Published:** 2025-11-19

**Authors:** Connor Willis, Andre Hejazi, Vonetta L. Williams, Craig Comperatore, Srinivas Tantravahi, Najla Al Ali, Jeffrey Gilreath, Tibor Kovacsovics, Trang Au, Malinda Tan, Madeline Brendle, Rami Komrokji, Minkyoung Yoo, Mushtaq Ashraf, Aamir Khakwani, Şerban R. Iorga, Islam Sadek, David A. Sallman, David Stenehjem

**Affiliations:** ^1^ Department of Pharmacotherapy, College of Pharmacy, University of Utah, Salt Lake City, Utah, USA, utah.edu; ^2^ H. Lee Moffitt Cancer Center & Research Institute, Tampa, Florida, USA, moffitt.org; ^3^ Huntsman Cancer Institute, University of Utah, Salt Lake City, Utah, USA, utah.edu; ^4^ Division of Epidemiology, Department of Internal Medicine, University of Utah, Salt Lake City, Utah, USA, utah.edu; ^5^ Novartis Pharmaceuticals, East Hanover, New Jersey, USA, novartis.com; ^6^ Department of Pharmacy Practice and Pharmaceutical Sciences, College of Pharmacy, University of Minnesota, Duluth, Minnesota, USA, umn.edu

## Abstract

This study describes treatment patterns and clinical and economic outcomes of patients with myelodysplastic syndrome (MDS) by Revised International Prognostic Scoring System (IPSS‐R) risk scores. A retrospective cohort study was conducted including adults diagnosed with MDS between 2010 and 2020 within two academic institutions. A total of 369 patients were included. Hypomethylating agents (HMAs) were used as first‐line therapy in 81% (*n* = 230) and 51% (*n* = 44) of higher‐risk (HR) and lower‐risk (LR) patients, respectively. Second‐line therapy was received by 24% of patients. Complete response was achieved by 11% of HR patients (median duration: 24 months). Median PFS and OS were 9.5 and 18.8 months in the HR cohort and 18.8 and 25.6 months in the LR cohort. Mean MDS‐related healthcare charges per patient per month were > 2‐fold higher among HR patients compared to LR (*p* < 0.001). An unmet need exists for first and subsequent lines of therapy.

**Summary**

• In the frontline treatment of higher‐risk MDS patients, there is a low likelihood of achieving/maintaining a complete response (CR) with high relapse rates.
• The MDS‐related healthcare charges were > 2‐fold higher among higher‐risk (HR) patients compared to lower‐risk (LR) patients.

## 1. Introduction

Myelodysplastic syndromes (MDSs) are a heterogeneous group of myeloid disorders characterized by ineffective hematopoiesis, cytopenia, and a risk of progression to acute myeloid leukemia (AML) [1, 2]. Symptoms can include fatigue, hemorrhage, or infections though many patients may be asymptomatic and diagnosed incidentally through routine blood screening in the early stages of the disease. Pathogenesis of MDS has been tied to somatic mutations in key genes involved in cell cycle regulation and certain chromosomal abnormalities [2, 3]. Diagnosis of MDS is based upon findings of a stable cytopenia with one of the following bone marrow findings: (1) dysplasia; (2) marrow blast cell counts 5%–19% (dependent on specific MDS morphology); or (3) cytogenetic/genetic findings [4]. The incidence of MDS in the US is approximately 4.5 per 100,000 per year among the general population, though incidence increases dramatically with age (55.4 per 100,000 per year for patients > 80 years) [1, 2]. Other risk factors for developing MDS include exposure to certain environmental factors, genetic abnormalities, and other benign hematologic diseases [5, 6]. MDS caused by prior exposure to cytotoxic drugs (notably alkylating agents and topoisomerase inhibitors) is known as therapy‐related or secondary MDS [4].

The Revised International Prognostic Scoring System (IPSS‐R) classifies disease risk based on bone marrow blast percentage, karyotype, hemoglobin, platelets (PLTs), and absolute neutrophil count [7]. Recently, a new scoring system for MDS known as the IPSS‐Molecular (IPSS‐M) has been published which incorporates a patient’s genetic profile into their IPSS‐R risk calculation [3, 8, 9]. The IPSS‐M is expected to help clarify the prognosis of patients with traditionally lower‐risk features and has shown promise in classifying patients with recently defined MDS/AML overlap [10, 11]. Current National Comprehensive Cancer Network (NCCN) guidelines propose the IPSS‐M be used as a part of the clinical decision‐making process but still defers to the IPSS‐R method for risk stratification [1].

Initial treatment of MDS is based on IPSS‐R risk scores, age, and other comorbidities. Treatment options fall into one of three categories: (1) supportive care; (2) low‐intensity therapy; or (3) high‐intensity therapy [4]. Supportive care for lower‐risk MDS patients consists of clinical monitoring, red blood cell or PLT transfusions, erythropoiesis‐stimulating agents (ESAs), and antibiotics or granulocyte colony‐stimulating factor (GCSF) support for infections [4]. Low‐intensity therapy typically consists of hypomethylating agents (HMAs): azacitidine or decitabine, whereas high‐intensity therapy, such as allogeneic hematopoietic cell transplant (allo‐HCT), is recommended for higher‐risk patients fit for such treatment [4].

Higher‐risk MDS is a disease with high unmet medical needs. A systematic review of higher‐risk MDS patients (including very HR, HR, and chronic myelomonocytic leukemia patients) identified ten studies that assessed treatment with HMAs and reported a range of median overall survival (OS) of 10.9–34 months [13]. One study reported a median PFS of 31.5 months [14]. A previous real‐world evidence study using the SEER‐Medicare database found the median OS to be 11.9 months, with a median time to AML transformation of 19.2 months among higher‐risk patients [15].

As the treatment landscape for patients with MDS is expected to evolve in the coming years, it will be important for healthcare stakeholders to understand the current and ongoing unmet clinical needs of these patients. Accurately defining these will inform both clinical practice and economic analyses. To this end, this study aims to describe the treatment patterns, outcomes, and economic impact of patients with MDS by IPSS‐R scores.

## 2. Materials and Methods

### 2.1. Study Design and Patient Population

This was a retrospective observational cohort study using patient‐level electronic health record (EHR) data from two—NCCN and National Cancer Institute (NCI)—designated comprehensive cancer centers in the U.S. Adult patients diagnosed with MDS between January 1, 2010, through December 31, 2020, were included in the study. MDS diagnosis was determined using relevant ICD‐9 or ICD‐10 coding (Supporting Table 1), and patients were required to have at least two MDS‐specific healthcare encounters at least 30 days apart within the study period. Additionally, patients were required to have received initial treatment for MDS at the study sites, based on their medication order history. Individuals enrolled in interventional/noninterventional MDS‐related clinical trials were included. Patients with a diagnosis of AML or a history of SCT prior to MDS diagnosis were excluded. Study protocol gained Institutional Review Board (IRB) approval at respective sites prior to initiation.

### 2.2. IPSS‐R Scoring and Baseline Characteristics

IPSS‐R scores were calculated for all included patients at MDS diagnosis (index date) using chart review from clinician documentation of individual score components (Supporting Table 2). Labs relevant for IPSS‐R calculation were collected ±30 days of index date. If multiple labs were available, the highest scoring values were utilized. Patients with a Very Low (≤ 1.5) and Low (> 1.5–3) IPSS‐R score were grouped into the LR cohort, and those with or Intermediate (> 3–4.5), High (> 4.5–6), or Very High (> 6) scores were grouped into the HR cohort.

Baseline demographics, comorbidities (Supporting Table 3), clinical characteristics, and genetic alteration data were collected from EHR databases for included patients.

### 2.3. Treatment Patterns

MDS‐related therapies received within the study period by line of therapy were identified by chart review. Therapies included HMAs (azacitidine and decitabine), cytarabine, antithymocyte globulin (ATG), lenalidomide, venetoclax, investigational drugs, and allo‐SCT.

### 2.4. Treatment Response and Clinical Outcomes

Treatment response was based on the International Working Group (IWG) response criteria for MDS [16]. Treatment response was categorized as CR defined as marrow blasts < 5% and hematologic recovery; marrow complete remission (mCR) defined as marrow blasts ≤ 5% and decrease by ≥ 50% over pretreatment; partial response (PR) defined as marrow blast decreased ≥ 50% but still > 5%; stable disease (SD) defined as failure to achieve at least a PR, but no evidence of progression for > 8 weeks; and progressive disease (PD) defined as an increase of ≥ 50% from baseline in marrow blasts or death.

Transfusion independence was defined as ≥ 8 weeks without packed red blood cell (pRBC) and/or PLT transfusion. Transfusion independence was evaluated in patients who required transfusion within 30 days prior to treatment initiation. Frequency and units of transfusions were evaluated by treatment patterns pre‐ and post‐treatment initiation (−30 days to +120 days).

OS was defined as the length of time from initiation of treatment for MDS (index date) until death (all‐cause); the observation period was censored at the date of last follow‐up. Information of patient death or last visit was obtained from electronic medical records and tumor registries. PFS was measured from initiation of treatment for MDS until progression to AML defined as marrow blasts greater than 20% [17] or physician documented treatment failure or death (all‐cause); the observation period was censored at the date of last follow‐up, initiation of 2L therapy, or time of transplant. By definition, only patients who received treatment for MDS were included in this analysis.

### 2.5. Economic Outcomes

All‐cause and MDS‐related healthcare charges were extracted from institutional hospital billing data. Regional charge data from the SEER registry were used to extrapolate these charges to all included patients. Charges were stratified by the number of inpatient (IP) and outpatient (OP) visits per patient per month (PPPM). The estimated yearly charges were adjusted for inflation to 2021 values using the Personal Consumption Expenditures price index for healthcare services [18].

### 2.6. Statistical Analysis

A descriptive analysis of study outcomes was conducted. Summary statistics, mean, and standard deviation (SD) were reported for continuous variables, and frequency and percentage were reported for categorical variables.

For evaluation of time‐to‐event variables, data from the date of treatment initiation to event date were used if available; otherwise, the observations were censored at the end of data availability. Survival analyses (PFS and OS) were conducted using Kaplan–Meier methodology from treatment initiation until death or censored at last follow‐up. PFS and OS were compared between LR and HR cohorts. Additionally, OS was compared by presence of common genetic alterations using a Cox regression analysis to generate a hazard ratio (HR) of mortality.

## 3. Results

**Figure 1:**
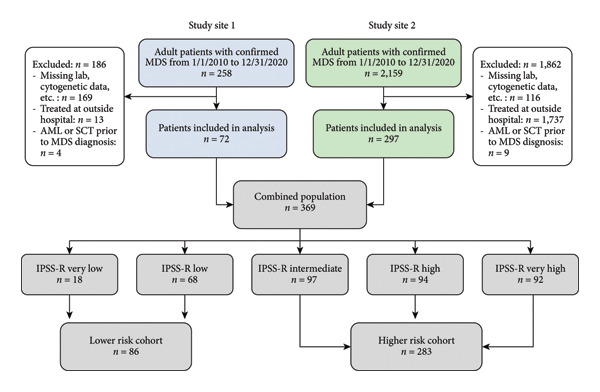
Study flow diagram.

A total of 369 patients were included in this study, with *n* = 86 (23%) in the LR cohort and *n* = 283 (77%) in the HR cohort. Patient selection flow diagram is shown in Figure 1.

### 3.1. Baseline Characteristics

Mean age for the entire cohort was 66 years, 56% (*n* = 208) were male, 93% (*n* = 342) were White, and 38% (*n* = 141) were transfusion independent at diagnosis (Tables 1 and 2). More patients had secondary MDS in the HR cohort compared to LR (25% vs. 17%, *p* = 0.003).

**Table 1:** Baseline characteristics.

Variables	LR (*n* = 86)	HR (*n* = 283)	All (*n* = 369)	*p* value^∗^
Age, mean (SD)	67.4 (10.9)	65.6 (10.5)	66.0 (10.6)	0.17
Age category, *n* (%)				
18–59	18 (21)	56 (20)	74 (20)	0.15
60–64	13 (15)	54 (19)	67 (18)	
65–69	15 (17)	67 (24)	82 (22)	
70–74	16 (19)	60 (21)	76 (21)	
≥ 75	24 (28)	46 (16)	70 (19)	
Unknown	NA	NA	NA	
Gender				
Male	48 (56)	160 (57)	208 (56)	0.91
Female	38 (44)	123 (43)	161 (44)	
Race/ethnicity, *n* (%)				
Caucasian/White	80 (93)	262 (93)	342 (93)	0.3
African American/Black	0 (0)	7 (2)	7 (2)	
Hispanic/Latino	1 (1)	2 (1)	3 (1)	
Native American	0 (0)	2 (1)	2 (1)	
Asian/Pacific Islander	2 (2)	6 (2)	8 (2)	
Other	3 (3)	2 (1)	5 (1)	
Unknown	0 (0)	2 (1)	2 (1)	
Insurance type, *n* (%)				
Commercial	28 (33)	115 (40)	143 (39)	0.38
Medicare	51 (59)	149 (53)	200 (54)	
Medicaid	2 (2)	7 (2)	9 (2)	
Uninsured	2 (2)	9 (3)	11 (3)	
Other/unknown	3 (3)	3 (1)	6 (2)	
Comorbidities, *n* (%)				
Any autoimmune disorder	9 (10)	24 (8)	33 (9)	0.55
Myocardial Infarction	8 (9)	23 (8)	31 (8)	0.73
Congestive heart failure	4 (5)	21 (7)	25 (7)	0.37
Peripheral vascular disease	7 (8)	15 (5)	22 (6)	0.33
Cerebrovascular disease	9 (10)	20 (7)	29 (8)	0.31
Chronic pulmonary disease	14 (16)	70 (25)	84 (23)	0.1
Dementia	1 (1)	3 (1)	4 (1)	0.94
Diabetes without complications	18 (21)	62 (22)	80 (22)	0.85
Diabetes with complications	7 (8)	21 (7)	28 (8)	0.83
Hemiplegia or paraplegia	1 (1)	1 (< 1)	2 (1)	0.37
Renal disease	18 (21)	54 (19)	72 (20)	0.71
Mild liver disease	7 (8)	65 (23)	72 (20)	0.002
Moderate/Severe liver disease	3 (3)	11 (4)	14 (4)	0.87
Peptic Ulcer disease	2 (2)	10 (4)	12 (3)	0.58
Rheumatologic disease	5 (6)	24 (8)	29 (8)	0.42
AIDS	1 (1)	0 (0)	1 (< 1)	0.07
None	35 (41)	81 (29)	116 (31)	0.04
BMI, mean (SD)	27.5 (4.4)	29.1 (6.4)	28.7 (6.0)	0.04
BMI category, *n* (%)				
< 18	0 (0)	4 (1)	4 (1)	0.02
18–24.9	24 (28)	77 (27)	101 (27)	
25–29.9	40 (47)	90 (32)	130 (35)	
> 30	20 (23)	110 (39)	130 (35)	
Unknown	2 (2)	2 (1)	4 (1)	

^∗^LR versus HR cohort.

**Table 2:** Cancer characteristics.

Variables	LR (*n* = 86)	HR (*n* = 283)	All (*n* = 369)	*p* value^∗^
WHO classification, *n* (%)				
MDS with single lineage dysplasia (MDS‐SLD), NOS	2 (2)	2 (1)	4 (1)	< 0.005
MDS with multilineage dysplasia (MDS‐MLD), NOS	21 (24)	34 (12)	55 (15)	
MDS with ring sideroblasts (MDS‐RS), NOS	9 (10)	8 (3)	17 (5)	
MDS‐RS‐SLD	3 (3)	1 (< 1)	4 (1)	
MDS‐RS‐MLD	6 (7)	12 (4)	18 (5)	
MDS with excess blasts (MDS‐EB), NOS	1 (1)	7 (2)	8 (2)	
MDS‐EB‐1	2 (2)	67 (24)	69 (19)	
MDS‐EB‐2	1 (1)	93 (33)	94 (25)	
MDS with isolated del (5q)	3 (3)	4 (1)	7 (2)	
MDS, unclassified (MDS‐U)	37 (43)	54 (19)	91 (25)	
Refractory cytopenia of childhood (provisional)	1 (1)	0 (0)	1 (< 1)	
Unknown	0 (0)	1 (< 1)	1 (< 1)	
ECOG performance status				
Grade 0	20 (23)	80 (28)	100 (27)	0.22
Grade 1	32 (37)	122 (43)	154 (42)	
Grade 2	7 (8)	11 (4)	18 (5)	
Grade ≥ 3	2 (2)	4 (1)	6 (1)	
Unknown	25 (29)	66 (23)	88 (25)	
Blast count at diagnosis, *n* (%)				
Mean blast count (SD)	1.9 (1.9)	7.7 (5.6)	6.4 (5.5)	< 0.005
Median blast count (IQR)				
≤ 2%	60 (70)	41 (14)	101 (27)	< 0.005
> 2% to < 5%	17 (20)	52 (18)	69 (19)	
5%–10%	3 (3)	105 (37)	108 (29)	
> 10%	1 (1)	78 (28)	79 (21)	
Unknown	5 (6)	7 (2)	12 (3)	
Cytogenetic risk category at diagnosis				
Very good	0 (0)	1 (0)	1 (0)	< 0.005
Good	66 (77)	103 (36)	169 (46)	
Intermediate	13 (15)	57 (20)	70 (19)	
Poor	3 (3)	27 (10)	30 (8)	
Very poor	0 (0)	90 (32)	90 (24)	
Unknown	4 (5)	5 (2)	9 (2)	
Absolute neutrophil count				
Median ANC (IQR)	1.7 (0.9–4.0)	1.1 (0.5–2.1)	1.2 (0.6–2.3)	0.57
≤ 0.8	15 (17)	88 (31)	103 (28)	0.047
> 0.8	53 (62)	145 (51)	198 (54)	
Unknown	18 (21)	50 (18)	68 (18)	
Hemoglobin				
Median hemoglobin (IQR)	9.9 (8.8–11.1)	9.2 (7.9–10.5)	9.4 (8.1–10.6)	0.01
≥ 10	39 (45)	95 (34)	134 (36)	0.003
8–< 10	34 (40)	113 (40)	147 (40)	
< 8	8 (9)	70 (25)	78 (21)	
Unknown	5 (6)	5 (2)	10 (3)	
Platelets				
Median platelets (IQR)	125 (78–283)	71 (37–120)	79 (42–141)	< 0.005
≥ 100	50 (58)	88 (31)	138 (37)	< 0.005
50–< 100	20 (23)	88 (31)	108 (29)	
< 50	11 (13)	101 (36)	112 (30)	
Unknown	5 (6)	6 (2)	11 (3)	
Secondary MDS				
Yes	15 (17)	72 (25)	87 (24)	0.003
No	66 (77)	209 (74)	275 (75)	
Unknown	5 (6)	2 (1)	7 (2)	
Transfusion independence at diagnosis				
Yes	35 (41)	106 (37)	141 (38)	0.01
No	40 (47)	164 (58)	204 (55)	
Unknown	11 (13)	13 (5)	24 (7)	

^∗^LR versus HR cohort.

Among LR patients with sequencing data (*n* = 81), *ASXL1* (23%), *SF3B1* (22%), *DNMT3A* (20%), *TP53* (12%), and *TET2* (12%) were the most frequently altered genes (Table 3). For HR patients (*n* = 267), *TP53* (35%), *ASXL1* (19%), *TET2* (19%), *RUNX1* (16%), and *DNMT3A* (14%) were most frequent. There was a higher rate of *TP53* mutations among the HR cohort (35% vs. 12%, *p* < 0.0005), while *SF3B1* mutations were more common in the LR cohort (22% vs. 5%, *p* < 0.0005).

**Table 3:** Genetic alterations.

	LR (*n* = 81)	HR (*n* = 267)	All (*n* = 348)	*p* value^∗^
Genetic alteration, *n* (%)				
TP53	8 (12)	78 (35)	86 (30)	< 0.005
ASXL1	15 (23)	43 (19)	58 (20)	0.51
TET2	8 (12)	42 (19)	50 (17)	0.22
DNMT3A	13 (20)	31 (14)	44 (15)	0.24
RUNX1	8 (12)	35 (16)	43 (15)	0.49
SRSF2	8 (12)	30 (13)	38 (13)	0.8
None	8 (12)	21 (9)	29 (10)	0.5
STAG2	4 (6)	22 (10)	26 (9)	0.35
SF3B1	14 (22)	11 (5)	25 (9)	< 0.005
Others	3 (5)	21 (9)	24 (8)	0.19
U2AF1	5 (8)	14 (6)	19 (7)	0.69
EZH2	6 (9)	10 (5)	16 (6)	0.14
BCOR	3 (5)	11 (5)	14 (5)	0.91
GATA2	3 (5)	9 (4)	12 (4)	0.84
IDH2	5 (8)	8 (4)	13 (5)	0.16
CEBPA	1 (2)	10 (5)	11 (4)	0.27
BCORL1	1 (2)	9 (4)	10 (3)	0.33
ZRSR2	2 (3)	8 (4)	10 (3)	0.84
ETV6	2 (3)	7 (3)	9 (3)	0.98
SETBP1	2 (3)	7 (3)	9 (3)	0.98
IDH1	2 (3)	6 (3)	8 (3)	0.87
JAK2	4 (6)	4 (2)	8 (3)	0.06
NRAS	2 (3)	6 (3)	8 (3)	0.87
Unknown	2 (3)	6 (3)	8 (3)	0.87
KMT2A	2 (3)	4 (2)	6 (2)	0.53
CUX1	1 (2)	4 (2)	5 (2)	0.89
PTPN11	2 (3)	3 (1)	5 (2)	0.35
WT1	0 (0)	5 (2)	5 (2)	0.22
FLT3	0 (0)	4 (2)	4 (1)	0.28
CBL	1 (2)	2 (1)	3 (1)	0.66
KDM6A	2 (3)	1 (< 1)	3 (1)	0.07
MPL	2 (3)	1 (< 1)	3 (1)	0.07
NOTCH1	1 (2)	2 (1)	3 (1)	0.66
NPM1	0 (0)	3 (1)	3 (1)	0.35
PHF6	1 (2)	2 (1)	3 (1)	0.66
ASXL2	0 (0)	2 (1)	2 (1)	0.44
CALR	0 (0)	2 (1)	2 (1)	0.44
CSF3R	0 (0)	2 (1)	2 (1)	0.44
ETNK1	1 (2)	1 (< 1)	2 (1)	0.35
KIT	0 (0)	2 (1)	2 (1)	0.44
KRAS	0 (0)	2 (1)	2 (1)	0.44
RAD21	0 (0)	2 (1)	2 (1)	0.44
USAF2	0 (0)	2 (1)	2 (1)	0.44
BCR‐ABL	0 (0)	1 (< 1)	1 (< 1)	0.59
BRAF	0 (0)	1 (< 1)	1 (< 1)	0.59
DDX41	0 (0)	1 (< 1)	1 (< 1)	0.59
DNMT1	0 (0)	1 (< 1)	1 (< 1)	0.59
GATA1	1 (2)	0 (0)	1 (< 1)	0.06
LUC7L2	1 (1)	0 (0)	1 (< 1)	0.06
PDGFR‐FIP1I1	0 (0)	1 (< 1)	1 (< 1)	0.59
SMC1A	0 (0)	1 (< 1)	1 (< 1)	0.59
SMC3	1 (2)	0 (0)	1 (< 1)	0.06
SUZ12	1 (2)	0 (0)	1 (< 1)	0.06

^∗^LR versus HR cohort.

### 3.2. Treatment Patterns

Treatment patterns for all patients are included in Table 4 and in Figure 2. The median time to treatment initiation after MDS diagnosis was 92 days (IQR 43‐212) for the LR cohort and 35 days (IQR 18‐67) for the HR cohort. Azacitidine was the predominant HMA used for 1L therapy among both study cohorts (LR: 45% [*n* = 39], HR: 73% [*n* = 206]). The median number of 28‐day HMA cycles was 5 and 4 for LR and HR cohorts, respectively. More patients were treated with lenalidomide as 1L therapy in the LR cohort (12% vs. 2%), and there was a higher percentage of patients not receiving any pharmacologic treatments in the LR cohort (26% vs. 11%). 2L therapy was received by 24% (*n* = 88) of patients across all groups (LR: 26% [*n* = 22], HR: 23% [*n* = 66]) with HMAs as the most frequently prescribed agent. Allo‐SCTs were performed in 49% (*n* = 180) of patients across all cohorts. More patients in the HR cohort received Allo‐SCT (LR: 27% [*n* = 23], HR: 56% [*n* = 159]) and were transplanted sooner after initial diagnosis (LR: 283 days, HR: 218 days).

**Table 4:** Treatment patterns.

	LR (*n* = 86)	HR (*n* = 283)	All (*n* = 369)
First‐line MDS therapy			
Median days to treatment initiation (IQR)	92 (43–212)	35 (18–67)	40 (20–82)
Azacitidine, *n* (%)	39 (45)	206 (73)	245 (66)
Decitabine, *n* (%)	5 (6)	24 (8)	29 (8)
HMA median cycles (IQR)	5 (4–13)	4 (3–7)	4 (3–7)
Cytarabine, *n* (%)	0 (0)	1 (< 1)	1 (< 1)
Antithymocyte globulin (ATG)	4 (5)	6 (2)	10 (3)
Lenalidomide, *n* (%)	10 (12)	6 (2)	16 (4)
Other^∗^, *n* (%)	6 (7)	8 (3)	14 (4)
None, *n* (%)	22 (26)	32 (11)	54 (15)
Second‐line MDS therapy			
Azacitidine, *n* (%)	9 (10)	27 (10)	36 (9)
Decitabine, *n* (%)	5 (6)	26 (9)	31 (8)
Cytarabine, *n* (%)	0 (0)	1 (0)	1 (< 1)
Venetoclax, *n* (%)	0 (0)	1 (0)	1 (< 1)
Antithymocyte globulin (ATG), *n* (%)	1 (1)	0 (0)	1 (< 1)
Lenalidomide, *n* (%)	4 (5)	3 (1)	7 (2)
Other^†^, *n* (%)	3 (3)	8 (3)	11 (3)
None, *n* (%)	64 (74)	217 (77)	281 (76)
Transplant			
Median days from diagnosis to transplant (IQR)	283 (157–457)	218 (156–288)	219 (157–295)
Stem‐cell transplant, *n* (%)	23 (27)	157 (55)	180 (49)
Post treatment, *n* (%)	16 (70)	140 (89)	156 (87)
During treatment, *n* (%)	0 (0)	1 (1)	1 (1)
Pretreatment, *n* (%)	1 (4)	3 (2)	4 (2)
No treatment, *n* (%)	6 (26)	13 (8)	19 (11)

^†^Clinical trial drug (*n* = 7), cladribine + cytarabine + G‐CSF + mitoxantrone (CLAG‐M), nivolumab, Inqovi, hydroxyurea, Revlimid, enasidenib, and eltrombopag.

^∗^Clinical trial drug (*n* = 5), hydroxyurea (*n* = 3), ruxolitinib (*n* = 2), rituximab (*n* = 2), ivosidenib, and methotrexate.

Figure 2(a) Treatment patterns for HR cohort. (b) Treatment patterns for LR cohort.

**Figure:**
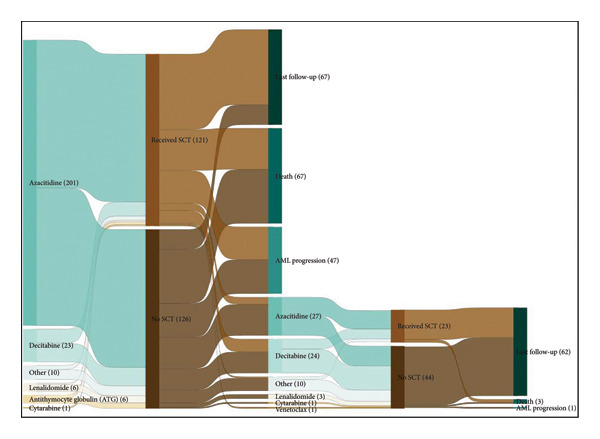
(a)

**Figure:**
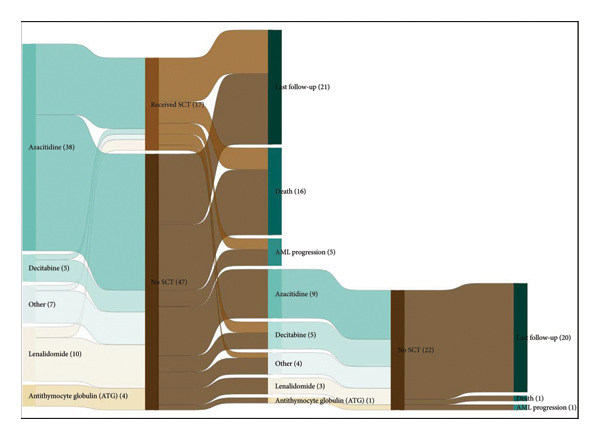
(b)

### 3.3. Treatment Response

Treatment response data for all included patients are included in Table 5. CR, partial remission (PR), and mCR following HMA therapy was achieved by 5% (*n* = 2), 2% (*n* = 1), and 0% (*n* = 0) of LR patients, compared to 11% (*n* = 26), 4% (*n* = 10), and 14% (*n* = 32) of HR patients, respectively. Stable disease was achieved by 57% (*n* = 25) of patients in the LR cohort and 43% (*n* = 98) of patients in the HR cohort, after treatment with an HMA. Best response after treatment initiation was achieved at 151 days and 109 days in the LR and HR cohorts, respectively.

**Table 5:** Treatment response.

1L treatment response	By 1L therapy			HMA‐treated	
	HMA (*n* = 274)	Other^∗^ (*n* = 41)	All (*n* = 315)	LR (*n* = 44)	HR (*n* = 230)
Complete response, *n* (%)	28 (10)	4 (10)	32 (10)	2 (5)	26 (11)
DoR, median months (IQR)	24 (13–33)	41 (22–78)	24 (14–35)	23 (12–34)	24 (14–33)
Marrow complete remission, *n* (%)	32 (12)	4 (10)	36 (11)	0 (0)	32 (14)
DoR, median months (IQR)	14 (10–26)	12 (9–15)	14 (10–25)	NA	14 (10–26)
Partial remission, *n* (%)	11 (4)	1 (2)	12 (4)	1 (2)	10 (4)
DoR, median months (IQR)	32 (22–46)	66 (NA)	35 (23–51)	32 (NA)	32 (22–46)
Stable disease, *n* (%)	123 (45)	15 (37)	138 (44)	25 (57)	98 (43)
Treatment failure, *n* (%)	2 (1)	0 (0)	2 (1)	0 (0)	2 (1)
Disease progression, *n* (%)	54 (20)	7 (17)	61 (19)	11 (25)	43 (19)
Relapse after CR/PR, *n* (%)	1 (< 1)	0 (0)	1 (< 1)	0 (0)	1 (< 1)
Death, *n* (%)	11 (4)	0 (0)	11 (3)	1 (2)	10 (4)
Other, *n* (%)	12 (4)	10 (24)	22 (7)	4 (9)	8 (3)
Time to best response^†^, median months (IQR)	4 (2–6)	5 (2–6)	4 (2–6)	5 (2–8)	4 (2–5)

Abbreviations: DoR, duration of response; HMA, hypomethylating agent; IQR, interquartile range.

^†^From initiation of 1L therapy.

^∗^Cytarabine, antithymocyte globulin (ATG), lenalidomide, hydroxyurea, ruxolitinib, rituximab, ivosidenib, methotrexate, or clinical trial drug.

### 3.4. Survival Outcomes

Kaplan–Meier curves for PFS and OS among patients receiving pharmacologic therapy (*n* = 315) are shown in Figure 3. Median PFS for the LR cohort (*n* = 64) was 18 months, versus 9 months for the HR cohort (*n* = 251). Median OS was 26 months for LR patients versus 19 months for HR patients.

Figure 3PFS and OS among treated patients (*n* = 315). (a) Progression‐free survival. (b) Overall survival.

**Figure:**
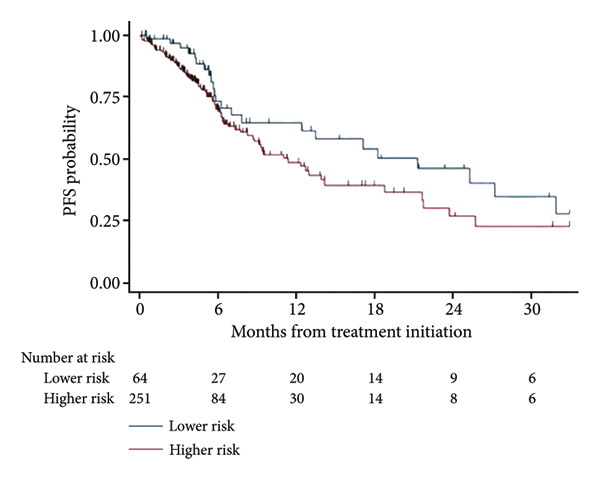
(a)

**Figure:**
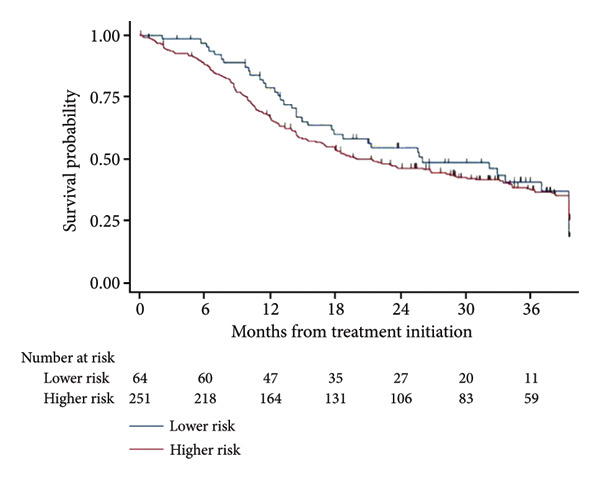
(b)

**Figure 4:**
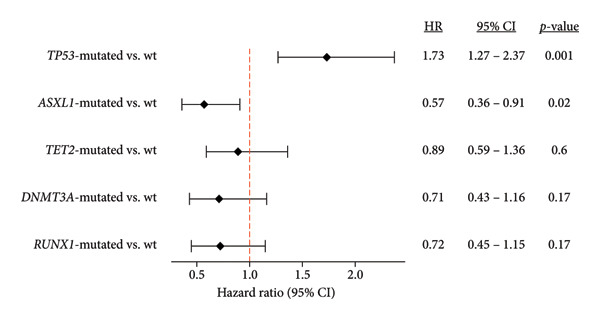
Hazard ratio (HR) for OS by key genomic alterations (*n* = 271).

OS in HMA‐treated patients (*n* = 230) within the HR cohort was compared according to the presence of the five most common genetic alterations within the cohort, *TP53* (*n* = 70), *ASXL1* (*n* = 33), *TET2* (*n* = 36), *RUNX1* (*n* = 32), and *DNMT3A* (*n* = 25). Median OS for *TP53*‐mutated patients was significantly shorter at 14 months compared to 27 months in wild‐type (wt) patients, with a hazard ratio (HR) of 1.70 (CI = 1.21–2.38, *p* = 0.002) (Figure 4). *ASXL1* mutations had a favorable prognosis, with significantly longer OS among *ASXL1*‐mutated patients (39 weeks) compared to wt (16 weeks) (HR = 0.46, CI = 0.26–0.82, *p* = 0.008).

### 3.5. Healthcare Charges

MDS‐related and all‐cause PPPM healthcare charges shown as group mean and SD are shown in Figure 5. For the overall cohort (*n* = 357), MDS‐related PPPM medical charges were $30,634 (SD: $71,403), with $11,376 ($43,981) from IP visits and $19,254 ($31,078) from OP visits. MDS‐related PPPM charges were > 2‐fold higher for the HR cohort (total medical: $35,148 ($80,617), IP: $13,303 ($49,799), and OP: $21,842 ($34,642), *p* < 0.001) compared to the LR cohort (total medical: $16,407 ($20,963), IP: $5301 ($13,220), and OP: $11,099 ($12,018); *p* < 0.001). All‐cause PPPM healthcare charges were > 2‐fold higher among HR patients compared to LR patients (Figure 5).

Figure 5PPPM healthcare charges. LR: Lower‐risk cohort, HR: Higher‐risk cohort. (a) MDS‐related. (b) All‐cause.

**Figure:**
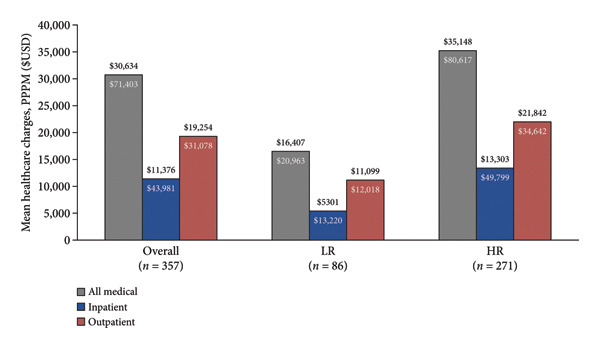
(a)

**Figure:**
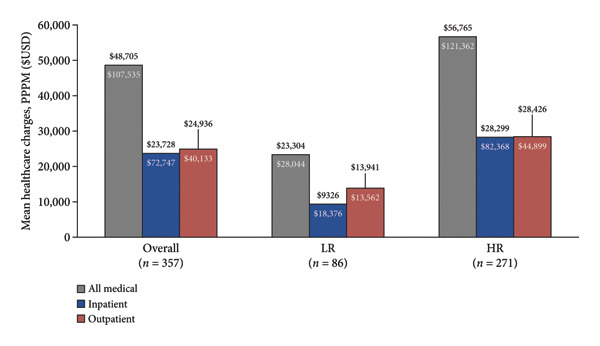
(b)

## 4. Discussion

In this retrospective observational cohort study, MDS patients at two—NCCN and NCI—designated comprehensive cancer centers were reviewed for treatment patterns, outcomes, and economic impact by IPSS‐R scores over a 10 year period. This study provides insights into real‐world practices for the treatment of patients with MDS and shows that the risk of death or AML transformation among patients with MDS was high despite HMA treatment, especially for those with higher‐risk MDS.

HMAs were frequently used as FL therapy in both the LR and HR cohorts. The median PFSs for patients in the HR and LR cohorts were 9 and 18 months, respectively. This time to progression to AML was shorter compared to those seen in other studies [14]. This could be due to the high rate of *TP53* mutations which can compromise the efficacy of chemotherapy agents, such as HMAs. The median OS was 19 and 26 months for the HR and LR cohorts, respectively, which are consistent with findings from previous studies [13, 15].

The IPSS‐M includes mutations in *TP53*, *FLT3*, and *KMT2A*‐*PTD* as the top predictors of adverse outcomes (PFS, OS, and AML transformation) in patients with MDS [8]. Mutations in *ASXL1, BCOR, EZH2, NRAS, RUNX1, STAG2,* and *U2AF1* were also significantly associated with adverse outcomes among these patients [8]. Our results observed a higher prevalence of SF3B1 in the LR cohort. SF3B1 has been shown to have better clinical outcomes and has been proposed as a distinct disease subtype by the IWG [20–22]. Additionally, the IPSS‐M study found that patients who were upstaged from intermediate to very high/high risk had two or more IPSS‐M adverse genes, suggesting that it is likely the cumulative contribution of each mutation that affects outcomes for these patients. Interestingly, the IPSS‐M study found an average PFS of 10 months for patients classified as IPSS‐R intermediate and IPSS‐M very high, which is similar to the findings of this study [8]. Further investigation on the use of recategorizing patients using the IPSS‐M method and the treatment outcomes should be considered as it may be more reflective of what is seen in real‐world patients.

In this study, we observed a higher rate of allo‐SCT among our population compared to previous studies, 49% overall, which could be attributed to several factors. Firstly, the inclusion criteria of this study mandated that patients receive some form of MDS therapy at academic medical centers affiliated with NCI/NCCN member institutions with robust transplant services. This ensured that the patients observed at these institutions had access to a high level of care and monitoring from the initiation of therapy for their MDS. Due to the resources available and expertise in SCT that these centers have, this could potentially lead to a greater likelihood of SCT treatment within our cohort. Additionally, specifying that patients must have received treatment in the inclusion criteria might have resulted in a population with more severe or refractory MDS cases, prompting a higher utilization of SCT as a therapeutic option.

Previous work has demonstrated PPPM costs for MDS patients comparable to the PPPM charges reported in this work [23, 24]. Our work highlighted the 2‐fold increase in PPPM charges for HR patients and the substantial proportion of charges that were related to out‐patient care (63%). Previous work has shown an increase in PPPM costs associated with transfusion dependence, comorbid disease burden, and HMA‐treatment failure [23, 24]. Previous studies have also reported outpatient costs as the main contributor (68%) of overall healthcare costs [24].

While this retrospective cohort analysis offers valuable insights into real‐world treatment of patients with MDS, this study also has several limitations. Relying on medical records for data collection makes this study susceptible to incomplete documentation and potential for miscoding of ICD‐9 or ICD‐10 codes leading to either inappropriate inclusion or exclusion of patients from the study. In addition, excluding patients who have not received treatment for MDS could lead to a population that has more severe or refractory MDS and less indolent cases. Furthermore, while academic medical centers offer specialized services and expertise, such as in SCT, the patient population treated and treatments offered may differ from those seen in community cancer centers, affecting the generalizability of the results of this study to the broader patient population. As multiple comparisons were included in this analysis, these results should be interpreted as hypothesis‐generating and should be verified in other datasets. Lastly, we were unable to calculate IPSS‐M scores for this patient cohort due to the absence of genetic details within the study dataset.

Strengths of this study include the multisite design that enabled us to capture a diverse patient population across different geographic regions and healthcare settings. Additionally, the extensive data collection spanning over a decade allowed for a comprehensive assessment of treatment patterns and outcomes in MDS patients. Lastly, despite the potential of miscoding with diagnostic codes, the use of diagnostic codes rather than relying solely on Medicare claims data increases the inclusivity of this study. It does so by encompassing individuals with and without Medicare insurance, thereby enhancing the generalizability of these results to a broader patient demographic.

In conclusion, the treatment patterns and outcomes for patients in the HR and LR cohorts show distinct differences. HR patients more frequently received HMAs as FL therapy and were more likely to undergo stem‐cell transplant (SCT) compared to LR patients. However, the likelihood of achieving a CR was low among HR patients, and their median progression‐free survival (PFS) and OS were notably shorter than those of LR patients. Additionally, healthcare costs were significantly higher for HR patients, indicating a substantial economic burden. These findings highlight an unmet need for more effective FL and subsequent therapies for HR patients to improve clinical outcomes and reduce healthcare costs.

## Ethics Statement

This study was reviewed and approved by the Institutional Review Board (IRB) at both the University of Utah (IRB# 00131223) and the Moffitt Cancer Center (IRB# 00000971).

## Disclosure

The content is solely the responsibility of the authors and does not necessarily represent the official views of the National Institutes of Health. All authors approved the final version of the paper to be published and agree to be accountable for all aspects of the work. The sponsor was involved in the design, analysis, and reporting of the study.

## Conflicts of Interest

Mushtaq Ashraf, Aamir Khakwani, Şerban R. Iorga, and Islam Sadek are or were employees of Novartis Pharmaceuticals. The other authors declare no conflicts of interest.

## Author Contributions

Conception and design: Connor Willis, Jeffrey Gilreath, Tibor Kovacsovics, Trang Au, Malinda Tan, Madeline Brendle, Islam Sadek, and David A. Sallman; data collection: Connor Willis, Vonetta L. Williams, Craig Comperatore, Trang Au, Malinda Tan, Madeline Brendle, David A. Sallman, and David Stenehjem; analysis and interpretation of the data: Connor Willis, Andre Hejazi, Vonetta L. Williams, Srinivas Tantravahi, Najla Al Ali, Jeffrey Gilreath, Tibor Kovacsovics, Malinda Tan, Madeline Brendle, Rami Komrokji, Minkyoung Yoo; Mushtaq Ashraf, Aamir Khakwani, Şerban R. Iorga, Islam Sadek, David A. Sallman, and David Stenehjem; drafting and revision of paper: all authors. David A. Sallman and David Stenehjem are co‐senior authors contributed equally to this manuscript. David A. Sallman and David Stenehjem are co‐senior authors and have contributed equally to this manuscript.

## Funding

This study was funded by Novartis Pharmaceuticals. This research was supported in part by the National Center for Advancing Translational Sciences of the National Institutes of Health under Award Number UL1TR002538. Connor Willis, Andre Hejazi, Vonetta L. Williams, Craig Comperatore, Najla Al Ali, Jeffrey Gilreath, Trang Au, Malinda Tan, Madeline Brendle, Rami Komrokji, Minkyoung Yoo, David A. Sallman, and David Stenehjem received research funding through the University of Utah or Moffitt Cancer Center from Novartis Pharmaceuticals to conduct this study.

## Supporting Information

The Supporting Information file contains 3 tables:

Table 1. ICD codes indicative of MDS.

This table provides a list of ICD‐9 and ICD‐10 codes used to identify patients with MDS.

Table 2. IPSS‐R cytogenic risk groups, prognostic score values, and prognostic risk categories/scores.

This table contains 3 subtables 1. Defines cytogenetic risk groups by cytogenetic abnormalities; 2. Defines the IPSS‐R scores by cytogenetics, bone marrow blast percentage, hemoglobin, platelets, and absolute neutrophil count; and 3. Defines IPSS‐R risk categories by IPSS‐R scores.

Table 3. ICD codes used in identifying comorbidities.

This table provides a list of ICD‐9 and ICD‐10 codes used to identify comorbidities.

## Supporting information


**Supporting Information** Additional supporting information can be found online in the Supporting Information section.

## Data Availability

The data that support the findings of this study are not publicly available due to privacy or ethical restrictions.
