# Supplementary material for: Treatment Patterns and Outcomes of Patients With Myelodysplastic Syndrome (MDS) by IPSS‐R Scores at Academic Cancer Centers
Source: Adv Hematol. 2025 Nov 19;2025:9924808. doi: 10.1155/ah/9924808 (PMC12629909; doi:10.1155/ah/9924808)
Supplement: Supplementary file 1 — Supporting Information Additional supporting information can be found online in the Supporting Information section. [file AH-2025-9924808-s001.pdf]

SUPPLEMENTARY MATERIALS

Supplementary Table 1. ICD codes indicative of MDS

**ICD codes indicative of MDS**	
ICD-9-CM	238.72, 238.73, 238.74, 238.75
ICD-10-CM	D46.0, D46.1, D46.2, D46.20, D46.21, D46.22, D46.A, D46.B, D46.C, D46.4, D46.Z, D46.9

Supplementary Table 2.: IPSS-R cytogenic risk groups, prognostic score values, and prognostic risk categories/scores

1. Cytogenic risk groups

**Cytogenetic prognostic subgroups**	**Cytogenetic abnormalities**
Very good	-Y, del(11q)
Good	Normal, del(5q), del(12p), del(20q), double including del(5q)
Intermediate	del(7q), +8, +19, i(17q), any other single or double independent clones
Poor	-7, inv(3)/t(3q)/del(3q), double including -7/del(7q), Complex: 3 abnormalities
Very poor	Complex: >3 abnormalities

2. Prognostic score values

**Prognostic variable**	**0**	**0.5**	**1**	**1.5**	**2**	**3**	**4**
Cytogenetics	Very good		Good		Intermediate	Poor	Very poor
BM blasts %	≤2		>2-<5		>5-<10	>10	
Hemoglobin	≥10		8-<10	<8			
Platelets	≥100	50-<100	<50				
ANC	≥0.8	<0.8					

3. Prognostic score risks

**Risk category**	**Risk score**
Very Low	<=1.5
Low	>1.5 - 3
Intermediate	>3 - 4.5
High	>4.5 - 6
Very High	>6

Supplementary Table 3. ICD codes used in identifying comorbidities

Comorbidities	ICD-10 codes	ICD-9 codes	Charlson Weight[19]
Myocardial infarction	I21.x, I22.x, I25.2	410.x, 412.x	1
Congestive heart failure	I09.9,I11.0, I13.0, I13.2, I25.5, I42.0, I42.5-I42.9, I43.x, I50.x, P29.0	398.91, 402.01, 402.11, 402.91, 404.01, 404.03, 404.11, 404.13, 404.91, 404.93, 425.4-425.9, 428.x	1
Peripheral vascular disease	I70.x, I71.x, I73.1, I73.8, I73.9, I77.1, I79.0, I79.2, K55.1, K55.8, K55.9, Z95.8, Z95.9	093.0, 437.3, 440.x, 441.x, 443.1-443.9, 447.1, 557.1, 557.9, V43.4	1
Cerebrovascular disease	G45.x, G46.x, H34.0, I60.x-I69.x	362.34, 430.x-438.x	1
Dementia	F00.x-F03.x, F05.1, G30.x, G31.1	290.x, 294.1, 331.2	1
Chronic pulmonary disease	I27.8, I27.9, J40.x-J47.x, J60.x-J67.x, J68.4, J70.1, J70.3	416.8, 416.9, 490.x-505.x, 506.4, 508.1, 508.8	1
Rheumatic disease	M05.x, M06.x, M31.5, M32.x-M34.x, M35.1, M35.3, M36.0	446.5, 710.0-710.4, 714.0-714.2, 714.8, 725.x	1
Peptic ulcer disease	K25.x-K28.x	531.x-534.x	1
Mild liver disease	B18.x, K70.0-K70.3, K70.9, K71.3-K71.5, K71.7, K73.x, K74.x, K76.0, K76.2-K76.4, K76.8, K76.9, Z94.4	070.22, 070.23, 070.32, 070.33, 070.44, 070.54, 070.6, 070.9, 570.x, 571.x, 573.3, 573.4, 573.8, 573.9, V42.7	1
Diabetes without chronic complication	E10.0, E10.l, E10.6, E10.8, E10.9, E11.0, E11.1, E11.6, E11.8, E11.9, E12.0, E12.1, E12.6, E12.8, E12.9, E13.0, E13.1, E13.6, E13.8, E13.9, E14.0, E14.1, E14.6, E14.8, E14.9	250.0-250.3, 250.8, 250.9	1
Diabetes with chronic complication	E10.2-E10.5, E10.7, E11.2-E11.5, E11.7, E12.2-E12.5, E12.7, E13.2-E13.5, E13.7, E14.2-E14.5, E14.7	250.4-250.7	2
Hemiplegia or paraplegia	G04.1, G11.4, G80.1, G80.2, G81.x, G82.x, G83.0-G83.4, G83.9	334.1, 342.x, 343.x, 344.0-344.6, 344.9	2
Moderate to severe renal disease	I12.0, I13.1, N03.2-N03.7, N05.2-N05.7, N18.x, N19.x, N25.0, Z49.0-Z49.2, Z94.0, Z99.2	403.01, 403.11, 403.91, 404.02, 404.03, 404.12, 404.13, 404.92, 404.93, 582.x, 583.0-583.7, 585.x, 586.x, 588.0, V42.0, V45.1, V56.x	2
Moderate or severe liver disease	I85.0, I85.9, I86.4, I98.2, K70.4, K71.1, K72.1, K72.9, K76.5, K76.6, K76.7	456.0-456.2, 572.2-572.8	3
AIDS/HIV	B20.x-B22.x, B24.x	042.x-044.x	6
